# Prognostic significance of troponin increment after percutaneous coronary intervention: A retrospective study

**DOI:** 10.3389/fcvm.2022.833522

**Published:** 2022-08-30

**Authors:** Ya Li, Duanbin Li, Liding Zhao, Tian Xu, Qingbo Lv, Jialin He, Yao Wang, Wenbin Zhang

**Affiliations:** ^1^Department of Cardiovascular Diseases, Sir Run Run Shaw Hospital, College of Medicine, Zhejiang University, Hangzhou, China; ^2^Key Laboratory of Cardiovascular Intervention and Regenerative Medicine of Zhejiang Province, Hangzhou, China

**Keywords:** periprocedural myocardial infarction, percutaneous coronary intervention, retrospective study, troponin, coronary artery disease

## Abstract

**Objective:**

The prognostic significance of troponin elevation following percutaneous coronary intervention (PCI) remains debated. This study aimed to evaluate the association between different thresholds of post-PCI cardiac troponin I (cTnI) and mortality.

**Methods:**

From January 2012 to July 2017, 5,218 consecutive patients undergoing elective PCI with pre-PCI cTnI < 99th percentile of the upper reference limit (URL) were included. Levels of cTnI were measured before PCI and every 8 h for 24 h after procedural. The outcomes were 3-year cardiac mortality.

**Results:**

Patients had a mean age of 66.2 years, 27.6% were women, 67.0% had hypertension, and 26.2% had diabetes mellitus. During the 3 years of follow-up, cardiac death occurred in 0.86%, 1.46%, 1.69%, 2.36%, and 2.86% of patients with cTnI < 1, ≥ 1 to < 5, ≥ 5 to < 35, ≥ 35 to < 70, and ≥ 70 times URL. The cardiac mortality rate was moderately increased with higher peak cTnI values, but the Kaplan–Meier curve demonstrated no significant association between any increment of cTnI and either cardiac or non-cardiac mortality. Isolated cTnI increment of ≥ 5 × URL, ≥ 35 × URL, and ≥ 70 × URL was occurred in 1,379 (26.4%), 197 (3.8%), and 70 (1.3%) patients, respectively. In multivariate Cox regression analysis and Fine-Gray model, none of the above cTnI thresholds was significantly associated with an increased risk of cardiac death.

**Conclusion:**

In patients who underwent elective PCI, post-PCI cTnI elevation is not independently associated with cardiac mortality.

## Introduction

More than 5 million patients each year are treated with percutaneous coronary intervention (PCI) worldwide (1). As the most frequent complication, the rate of periprocedural myocardial infarction (PMI) remains high, with potentially worse long-term outcomes (2). Biomarkers, such as creatine kinase MB (CK-MB) and cardiac troponin (cTn), have been used to diagnose PMI. However, the peak biomarker thresholds that define PMI markedly vary across different guidelines (3–7). This includes the Third (3) and Fourth (4) Universal Definition of Myocardial Infarction (UDMI) criteria, Academic Research Consortium-2 (ARC-2) (5), and the Society for Cardiovascular Angiography and Interventions (SCAI) (6). Several studies have shown that increment of post-PCI CK-MB is independently predictive of poor prognosis (8–11). However, the european society of cardiology (ESC) Study Group recommended that CK-MB is no longer needed to detect PMI (12). Although cTn is more sensitive than CK-MB, adopting a troponin-based definition of PMI remains debatable because of its uncertain prognostic significance (2, 8, 13, 14). Currently, the cutoff thresholds of post-PCI cTn elevation that define PMI are based on expert consensus opinion (15).

The present study explored whether different thresholds of cTn elevation after elective PCI could predict 3-year cardiac mortality in coronary artery disease (CAD) patients with pre-PCI cTn values < 99th percentile upper reference limit (URL).

## Materials and methods

### Study population

This was a retrospective single-center study. Consecutive patients treated with elective PCI from January 2012 to July 2017 with pre-PCI cardiac troponin I (cTnI) levels < 99th percentile URL were screened at Sir Run Run Shaw Hospital of Zhejiang University. Each patient was included in the analysis only once (i.e., at the patient’s first PCI procedure in our hospital). The inclusion criteria were (1) patients who received elective successful stent implantation; (2) patients with pre-PCI cTnI and CK-MB levels < 99th percentile URL, and available cTnI measurements every 8 h for 24 h post-PCI; and (3) patients with complete 3-year follow-up.

Exclusion criteria included (1) patients experienced MI 30-day prior to elective PCI; (2) patients had chronic total occlusion whether the lesions would be opened or not; and (3) loss to follow-up within 3 years after PCI.

The study was conducted with strict adherence to the ethical principles outlined in the Declaration of Helsinki (as revised in 2013) and was approved by the ethics review committee at Sir Run Run Shaw Hospital (no. KY20201217-36).

### Biochemical measurements

Blood samples for cTnI measurements were obtained before PCI (at admission) and every 8 h after PCI (usually 24 h), and the highest values of post-PCI cTnI would be collected for analysis. Standard cTnI was measured using the enzyme-linked fluorescent assay (VIDAS Troponin I Ultra, bioMérieux). The 99th percentile URL for this measurement was 0.11 ng/ml. For this analysis, the different thresholds of peak cTnI were formulated according to the isolated myocardial biomarker criterion for the 4th UDMI definition (4), ARC-2 (5), and SCAI (6) (cTnI ≥ 5 × URL, ≥ 35 × URL, and ≥ 70 × URL, respectively).

### Endpoint

The outcomes were all-cause, cardiac, and non-cardiac mortality during 3 years of follow-up after PCI. Follow-up was performed by telephone contact yearly after PCI.

### Statistical analysis

Statistical analysis was conducted using SPSS version 22.0 (Chicago, IL, United States) and Stata 17.0 (TX, United States). Continuous variables were expressed as mean ± SD for variables with normal distribution or median (interquartile range) for variables with skewed distribution. Categorical variables were presented as frequencies.

Cardiac mortality and non-cardiac mortality of CAD patients with normal baseline myocardial biomarkers were compared among patients stratified by commonly used cTnI intervals (< 1, ≥ 1 to < 5, ≥ 5 to < 35, ≥ 35 to < 70, and ≥ 70 × URL). Event rates were calculated using the Cox model and were compared using the log-rank test. When cardiac death and non-cardiac death occurred as competitive events, the Fine-Gray model was performed for analysis. Adjusted hazard ratios (HRs) and 95% confidence intervals (CIs) for cardiac mortality and non-cardiac mortality were calculated by multivariable Cox regression analysis to compare groups as follows: cTnI < 5 vs. ≥ 5 × URL, cTnI < 35 vs. ≥ 35 × URL, and cTnI < 70 vs. ≥ 70 × URL. All *p*-values were 2-sided, and statistical significance was set at *p* < 0.05.

## Results

### Patient characteristics

**FIGURE 1:**
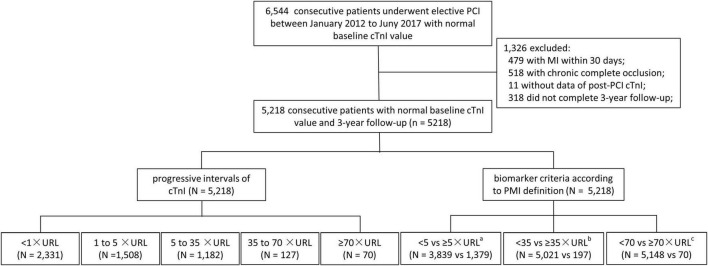
Study flowchart. From January 2012 to July 2017, 6,544 consecutive patients underwent PCI with normal baseline cTnI value. After excluding 11 patients with baseline increased or missing post-PCI cTnI values, 479 within 30-day MI, 518 with chronic complete occlusion, and 318 patients who were lost to follow-up, 5,218 consecutive patients with 3-year follow were finally included in this analysis. cTnI, cardiac troponin I; MI, myocardial infarction; PCI, percutaneous coronary intervention; URL, upper reference limit. ^a^According to biomarker criteria of PMI defined by third and fourth universal definition of myocardial infarction criteria; ^b^according to biomarker criteria of PMI defined by Academic Research Consortium-2; ^c^according to biomarker criteria of PMI defined by the Society for Cardiovascular Angiography and Interventions.

The design of the present study is shown in Figure 1. After excluding 11 patients with missing post-PCI cTnI values, 479 within 30-day MI, 518 with chronic complete occlusion, and 318 patients who were lost to follow-up, 5,218 consecutive patients with 3 years of follow-up were finally included in this analysis. All included patients underwent elective PCI from January 2012 to July 2017 and had normal baseline cTnI and 3 years of follow-up.

The key demographic data, angiographic features, and procedural characteristics of the patients are listed in Table 1. As shown, patients had a mean age of 66.2 years, 27.6% were women, 67.0% had hypertension, and 26.2% had diabetes mellitus. Most of the patients had a one-vessel disease (74.9%). The left anterior descending CAD was involved in 68.8% of the patients, with moderate or severe complexity lesions (type B2/C) present in 68.5% of patients. Stents with a median length of 36 mm per patient were implanted.

**TABLE 1:** Baseline clinical characteristics, angiographic characteristics, and procedural characteristics of the study population (*N* = 5,218).

Clinical characteristics	
Age (years), mean ± SD	66.2 ± 10.4
Female, n (%)	1,441 (27.6)
BMI (kg/m^2^), mean ± SD	24.6 ± 6.4
Current smoking, n (%)	1,173 (22.5)
Diabetes, n (%)	1,366 (26.2)
Hypertension, n (%)	3,494 (67.0)
Prior stroke, n (%)	452 (8.7)
Prior MI, n (%)	410 (7.9)
Prior PCI, n (%)	1,264 (24.2)
Perioperative medications, n (%)	
Clopidogrel	4,743 (90.9)
Ticagrelor	372 (7.1)
ACEI/ARB	2,955 (56.6)
Beta-blocker	2,670 (50.2)
Calcium-channel blocker	1,647 (31.6)
eGFR (mL/min/1.73 m^2^)	79.6 ± 28.9
LDL-C (mmol/L), mean ± SD	2.1 ± 0.88
LVEF (%), mean ± SD	65.1 ± 9.4
**Angiographic characteristics**	
Lesions of vessels, n (%)	
Left main	563 (10.8)
Left anterior descending	3,590 (68.8)
Left circumflex	1,511 (29.0)
Right coronary artery	1,981 (38.0)
Number of diseased coronary arteries, n (%)	
1	3,909 (74.9)
2	1,219 (23.4)
3	90 (1.7)
AHA/ACC classification B_2_/C, n (%)	3,574 (68.5)
Calcification, n (%)	854 (16.4)
**Procedural characteristics, techniques**	
FFR/IVUS/OCT, n (%)	745 (14.3)
Total stent length per patient (mm), median (IQR)	36 (23,58)
Mean stent size > 2.5 mm, n (%)	4,520 (86.6)
Balloon pre-dilation, n (%)	4,459 (91.6)
Balloon post-dilation, n (%)	4,778 (91.6)
Rotational ablation, n (%)	67 (1.3)
Number of patients with cTnI above threshold, n (%)	
≥ 1 × URL	2,887 (55.3)
≥ 3 × URL	1,803 (34.6)
≥ 5 × URL	1,379 (26.4)
≥ 10 × URL	739 (14.2)
≥ 20 × URL	380 (7.3)
≥ 35 × URL	197 (3.8)
≥ 70 × URL	70 (1.3)

Data are presented as mean ± SD or % (n/N). ACEI, angiotensin-converting enzyme inhibitors; AHA/ACC, American Heart Association/American College of Cardiology; ARB, angiotensin receptor blocker; BMI, body mass index; eGFR, estimated glomerular filtration rate; FFR, fractional flow reserve; IQR, interquartile range; IVUS, intravascular ultrasound; LDL-C, low-density lipoprotein cholesterol; LVEF, left ventricular ejection fraction; MI: myocardial infarction; OCT, optical coherence tomography; PCI, percutaneous coronary intervention; SD, standard deviation; URL, upper reference limit.

### Post-PCI cTnI result

The frequencies of cTnI elevations by different cutoff values varied considerably (Table 1). The occurrence of post-PCI cTnI increments was 55.3%, and cTnI ≥ 3 ×, ≥ 5 ×, ≥ 10 ×, ≥ 35 ×, and ≥ 70 × URL occurred in 1,803 (34.6%), 1,379 (26.4%), 739 (14.2%) 197 (3.8%), and 70 (1.3%) of cases.

### Post-PCI cTnI and mortality

**FIGURE 2:**
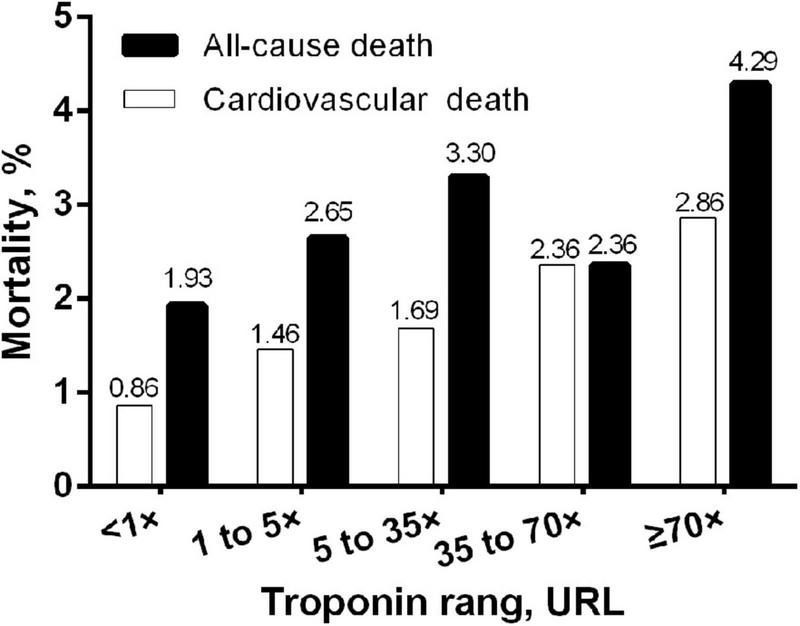
Bar graph of 3-year mortality rate by different ranges of post-PCI peak cTnI normalized to the upper reference limit. URL, upper reference limit; cTnI, cardiac troponin I.

During the 3-year follow-up, 130 (2.49%) all-cause mortality events occurred, of which 67 (1.28%) were of cardiovascular mortality. Figure 2 shows increasing mortality across intervals of troponin increment and the 3-year cardiovascular mortality stabilizes for troponin intervals.

**FIGURE 3:**
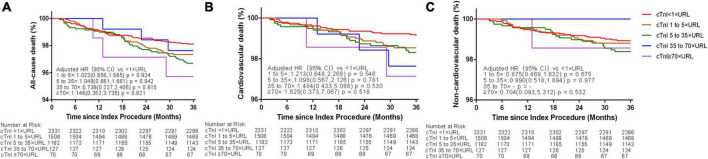
Three-year Kaplan–Meier curves for all-cause **(A)**, cardiovascular **(B)**, and non-cardiovascular death **(C)** according to different thresholds of post-PCI peak cTnI values in patients with elective PCI. URL, upper reference limit; cTnI, cardiac troponin I.

Kaplan–Meier curves for cardiac and non-cardiac deaths showed no significant difference between the 5 groups (log-rank *p* for cardiac death = 0.521; log-rank *p*-value for non-cardiac death = 0.532, Figure 3). In the multivariate Cox regression analysis, the cTn ratio was not an independent predictor of cardiac or non-cardiac death after adjustment for age, gender, hypertension, diabetes mellitus, current smoking, low-density lipoprotein cholesterol, glomerular filtration rate, left ventricular ejection fraction, prior MI, number of diseased vessels, the complexity of lesions, and total stented length (*p*-value for all > 0.05, Table 2). The multivariate Fine-Gray model indicated that cTn ratios were not independently associated with cardiac death [subdistribution HR (SHR) 1.101, 95% CI 0.865–1.403, *p* = 0.433].

**TABLE 2:** Hazard ratios for 3-year mortality with different thresholds of post-PCI peak cTn values.

Event	%(n/N)	Unadjusted HR (95% CI)	*P-value*	Adjusted HR (95% CI)	*P-value*
**All-cause mortality**					
< 1 × URL	1.93 (45/2888)	Reference	–	Reference	–
1–5 × URL	2.65 (40/1508)	1.378 (0.900, 2.109)	0.140	1.022 (0.658, 1.585)	0.924
5–35 × URL	3.30 (39/1182)	1.717 (1.118, 2.636)	0.014	1.048 (0.661, 1.661)	0.842
35–70 × URL	2.36 (3/127)	1.220 (0.379, 3.927)	0.738	0.738 (0.227, 2.406)	0.615
≥ 70 × URL	4.39 (3/70)	2.242 (0.697, 7.213)	0.176	1.146 (0.352, 3.735)	0.821
*P* value for trend			0.014		0.961
**Cardiovascular mortality**					
< 1 × URL	0.86 (20/2888)	Reference	–	Reference	–
1–5 × URL	1.46 (22/1508)	1.704 (0.930, 3.122)	0.084	1.213 (0.648, 2.268)	0.546
5–35 × URL	1.69 (20/1182)	1.979 (1.065, 3.678)	0.031	1.098 (0.567, 2.126)	0.781
35–70 × URL	2.36 (3/127)	2.749 (0.817, 9.252)	0.102	1.484 (0.433, 5.088)	0.530
≥ 70 × URL	2.86 (2/70)	3.355 (0.784, 14.352)	0.103	1.625 (0.373, 7.067)	0.518
*P* value for trend			0.007		0.521
**Non-cardiovascular mortality**					
< 1 × URL	0.87 (25/2888)	Reference	–	Reference	–
1–5 × URL	1.19 (18/1508)	1.114 (0.608, 2.042)	0.727	0.875 (0.469, 1.632)	0.675
5–35 × URL	1.61 (19/1182)	1.500 (0.826, 2.724)	0.183	0.990 (0.518, 1.894)	0.977
35–70 × URL	0.00 (0/127)	–	–	–	–
≥ 70 × URL	1.43 (1/70)	1.336 (0.181, 9.857)	0.777	0.704 (0.093, 5.312)	0.734
*P* value for trend			0.477		0.532

Covariates used in the adjusted model include age, gender, hypertension, diabetes mellitus, current smoking, low-density lipoprotein cholesterol, glomerular filtration rate, left ventricular ejection fraction, prior MI, number of diseased vessels, complexity of lesions, and total stented length. URL, upper reference limit.

**FIGURE 4:**
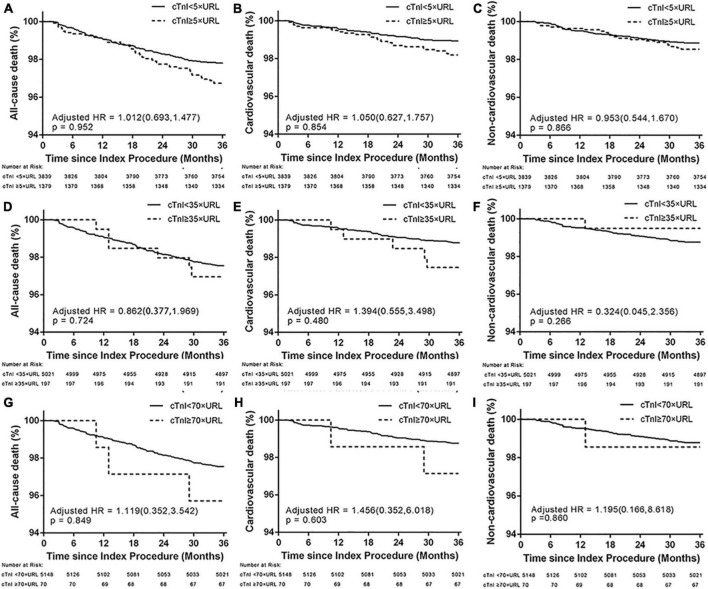
Three-year Kaplan–Meier curves for all-cause death **(A,D,G)**, cardiovascular **(B,E,H)**, and non-cardiovascular death **(C,F,I)** in patients who underwent PCI with different post-PCI peak cTnI values according to the biomarker criterion of the 4th UDMI, ARC-2, and SCAI definitions. ARC-2, Academic Research Consortium-2; SCAI, Society for Cardiovascular Angiography and Interventions; UDMI, Universal definition of myocardial infarction; URL, upper reference limit.

Cardiac troponin I ratios that have been proposed according to biomarker criteria and according to the 4th UDMI, ARC-2, and SCAI definitions were cTnI ≥ 5 ×, ≥ 35 ×, and ≥ 70 × URL. After adjustment for age, gender, hypertension, diabetes mellitus, current smoking, low-density lipoprotein cholesterol, glomerular filtration rate, left ventricular ejection fraction, prior MI, the number of diseased vessels, the complexity of lesions, and total stented length, none of the various myocardial injuries defined by different isolated post-cTnI values was significantly associated with the rate of cardiac mortality or non-cardiac mortality (*p*-value for all > 0.05, Table 3 and Figure 4). The multivariate Fine-Gray model indicated that cTnI ratios ≥ 5 ×, ≥ 35 ×, and ≥ 70 × URL were not associated with cardiac death (cTnI ≥ 5 × URL: SHR 1.304, 95% CI 0.767–2.216, *p* = 0.327; cTnI ≥ 35 × URL: SHR 0.415, 95% CI 0.057–3.006, *p* = 0.384; cTnI ≥ 70 × URL: SHR 1.212, 95% CI 0.166–8.843, *p* = 0.850).

**TABLE 3:** Hazard ratios for 3-year mortality in patients with different post-PCI peak cTnI values according to the biomarker criterion of the 4th UDMI, ARC-2, and SCAI definitions.

Event	% (n/N)	% (n/N)	Unadjusted HR (95% CI)	*P-value*	Adjusted HR (95% CI)	*P-value*
**All-cause mortality**						
< 5 × URL vs. ≥ 5 × URL	2.21 (85/3839)	3.26 (45/1379)	1.478 (1.035, 2.259)	0.033	1.012 (0.693, 1.477)	0.952
< 35 × URL vs. ≥ 35 × URL	2.47 (124/5021)	3.05 (6/197)	1.233 (0.511, 3.105)	0.616	0.862 (0.377, 1.969)	0.724
< 70 × URL vs. ≥ 70 × URL	2.47 (127/5148)	4.29 (3/70)	1.751 (0.469, 9.429)	0.944	1.119 (0.353, 3.542)	0.849
**Cardiovascular mortality**						
< 5 × URL vs. ≥ 5 × URL	1.09 (42/3839)	1.81 (25/1379)	1.661 (1.020, 3.024)	0.042	1.05 (0.627, 1.757)	0.854
< 35 × URL vs. ≥ 35 × URL	1.23 (62/5021)	2.54 (5/197)	2.062 (0.789, 9.771)	0.112	1.394 (0.555, 3.498)	0.480
< 70 × URL vs. ≥ 70 × URL	1.26 (65/5148)	2.86 (2/70)	2.276 (0.435, 28.28)	0.239	1.456 (0.352, 6.018)	0.603
**Non-cardiovascular mortality**						
< 5 × URL vs. ≥ 5 × URL	1.12 (43/3839)	1.45 (20/1379)	1.295 (0.762, 2.201)	0.340	0.953 (0.544, 1.670)	0.866
< 35 × URL vs. ≥ 35 × URL	1.23 (62/5021)	0.51 (1/197)	0.410 (0.057, 2.955)	0.376	0.324 (0.045, 2.356)	0.266
< 70 × URL vs. ≥ 70 × URL	1.20 (62/5148)	1.43 (1/70)	1.189 (0.165, 8.574)	0.864	0.761 (0.104, 5.561)	0.788

Covariates used in the adjusted model include age, gender, hypertension, diabetes mellitus, current smoking, low-density lipoprotein cholesterol, glomerular filtration rate, left ventricular ejection fraction, prior MI, number of diseased vessels, complexity of lesions, and total stented length. ARC-2, Academic Research Consortium-2; SCAI, Society for Cardiovascular Angiography and Interventions; UDMI, Universal definition of myocardial infarction; URL, upper reference limit.

## Discussion

In this large-scale study that included more than 5,000 consecutive patients who underwent PCI, isolated post-PCI cTnI of any cutoff showed no significant association with cardiovascular mortality.

### Comparison with previous studies

There is a debate regarding the prognostic significance of cTnI elevations after PCI. On the one hand, numerous studies and meta-analyses have noted a significant association between an increment of post-PCI cTn and mortality (13, 16–20). A large meta-analysis of 44,972 chronic patients with coronary syndrome who underwent PCI from 24 prospective studies demonstrated that cTn levels > 3 × URL was associated with increased mortality at 1 year [odds ratio (OR) 1.51, 95% CI 1.05–2.17]. However, this meta-analysis did not adjust for other factors affecting the outcomes (16). In a more recently patient-level pooled analysis of 9,081 patients who underwent PCI, cTn levels ≥ 5 × URL were strongly associated with 1-year mortality with an adjusted OR of 2.29 (19). On the other hand, in contrast to these studies, there have been reports showing no prognostic information from post-PCI cTn (21–23). In a pooled analysis that included 13,038 patients with ACS who underwent PCI from 2 trials, Tricoci et al. (9) did not find an increased mortality rate with a isolated cTn ratio > 5 × URL or even > 10 × URL. In addition, several studies reported that cTn elevation ≥ 70 × URL, which was required as an SCAI criterion, did not increase the risk of adverse outcomes (8, 14). In a pooled analysis of 13,452 patients from 5 trials and 1 registry, Hector et al. (14) showed neither significant association between cTn elevations at any degree and increased mortality nor between cTn elevation ≥ 70 × URL with 1-year mortality (HR 1.628, 95% CI 0.88–3.00, *p* = 0.120). Similarly, in a recent study of 4,031 patients who underwent left main PCI, no level of post-PCI cTn elevations was associated with cardiac or all-cause mortality, even for ≥ 70 × URL (8). Our results are consistent with these studies showing no increase in cardiac and all-cause mortality irrespective of cTn increment at any cutoff even when cTn increment is ≥ 70 × URL.

Based on the results of our study, PCI-related MI defined solely on cTn elevations may be inappropriate. This could be because cTn is too oversensitive to define PCI-related myocardial damage. While as another cardiac biomarker, CK-MB has shown a strong association with adverse outcomes in many previous studies (8–10). Furthermore, the combination of additional evidence of coronary ischemia improved the prognostic significance, compared with isolated cTn elevations. Tricoci et al. observed a twofold increase in mortality among patients with cTn elevations and clinical or angiographic complications, whereas isolated cTn ≥ 5 × or ≥ 10 × URL increment was not associated with mortality (9).

### Limitations

Several limitations should be noted. First, because of the retrospective nature of our study, only biomarker data were available, while data on ischemic symptoms (i.e., chest pain after PCI), electrocardiographs, and angiograms were hard to collect. Thus, this study is aimed to explore the long-term prognostic significance of isolated post-PCI cTnI elevation but not the prognostic significance of PMI. Second, although we adjusted for several potential confounders in the models, residual confounding due to unmeasured or unknown factors remains possible. For example, plaque characteristics was not accessible in this study because intravascular ultrasound/optical coherence tomography (IVUS/OCT) were only used in part of patients. Third, patients with increased pre-PCI cardiac biomarkers were not included in the study. Thus, our results are generalizable to patients with a normal baseline only. Similarly, our findings apply to standard cTn only but not to high-sensitivity cTn.

## Conclusion

In patients with CAD who underwent elective PCI, post-PCI cTn elevations of any degree were not predictive of cardiac mortality in the absence of procedural complications or evidence of new myocardial ischemia.

## Data availability statement

The original contributions presented in this study are included in the article/supplementary material, further inquiries can be directed to the corresponding author.

## Ethics statement

The studies involving human participants were reviewed and approved by the Ethics Review Committee at Sir Run Run Shaw Hospital. Written informed consent for participation was not required for this study in accordance with the national legislation and the institutional requirements.

## Author contributions

YL and DL reviewed the literature and contributed to the manuscript drafting. LZ, TX, QL, JH, and YW contributed to the data collection, interpretation, and analysis. WZ was responsible for conception, design, and administrative support. All authors issued final approval for the version to be submitted.
